# Regulatory roles of ferroptosis-related non-coding RNAs and their research progress in urological malignancies

**DOI:** 10.3389/fgene.2023.1133020

**Published:** 2023-03-02

**Authors:** Shijin Wang, Bowen Jiang, Deqian Xie, Xiunan Li, Guangzhen Wu

**Affiliations:** Department of Urology, The First Affiliated Hospital of Dalian Medical University, Dalian, China

**Keywords:** ferroptosis, non-coding RNAs, urological oncology, bladder cancer, prostate cancer, kidney cancer

## Abstract

Ferroptosis is a new type of cell death characterized by damage to the intracellular microenvironment, which causes the accumulation of lipid hydroperoxide and reactive oxygen species to cause cytotoxicity and regulated cell death. Non-coding RNAs (ncRNAs) play an important role in gene expression at the epigenetic, transcriptional, and post-transcriptional levels through interactions with different DNAs, RNAs, or proteins. Increasing evidence has shown that ferroptosis-related ncRNAs are closely related to the occurrence and progression of several diseases, including urological malignancies. Recently, the role of ferroptosis-associated ncRNAs (long non-coding RNAs, micro RNAs, and circular RNAs) in the occurrence, drug resistance, and prognosis of urological malignancies has attracted widespread attention. However, this has not yet been addressed systematically. In this review, we discuss this issue as much as possible to expand the knowledge and understanding of urological malignancies to provide new ideas for exploring the diagnosis and treatment of urological malignancies in the future. Furthermore, we propose some challenges in the clinical application of ferroptosis-associated ncRNAs.

## 1 Introduction

In 2018, the Nomenclature Committee on Cell Death categorized cell death mechanisms into regulated cell death (RCD) and accidental cell death (Galluzzi et al., 2018). RCD is an autonomous and orderly cell death strictly regulated by genes that maintain homeostasis of the internal environment, which is essential for the growth and development of the body, maintenance of homeostasis, and development of disease (Koren and Fuchs, 2021; Peng et al., 2022). The abnormal proliferation of tumor cells and evasion of cell death are important biological features of malignant tumors. These features plausibly explain the lethality, aggressive metastasis, and treatment resistance of tumors (Hanahan and Weinberg, 2011). Ferroptosis is a new form of RCD characterized by the accumulation of reactive oxygen species (ROS) and depletion of polyunsaturated fatty acids in the plasma membrane when the intracellular environment is specifically disturbed, ultimately leading to iron-dependent oxidative cell death. Its morphological, genetic, and biochemical manifestations differ from those of autophagy, apoptosis, necrosis, and pyroptosis (Dixon et al., 2012; Yang and Stockwell, 2016; Stockwell et al., 2017; Sun et al., 2022a).

Non-coding RNAs (ncRNAs) can be roughly classified into microRNAs (miRNAs), long ncRNAs (lncRNAs), and circular RNAs (circRNAs) according to their length and morphology (Adams et al., 2017; Yan and Bu, 2021). With the widespread application of high-throughput sequencing technology, many ncRNAs have been found to be involved in the pathophysiological processes of multiple diseases, and previously unappreciated ncRNAs have been revitalized (ENCODE Project Consortium, 2012; Djebali et al., 2012). Furthermore, ncRNAs have been found to play significant roles in various diseases, including tumors (Slack and Chinnaiyan, 2019; Zhang et al., 2020a; Wen et al., 2020). Generally, miRNAs are endogenous single-stranded small molecule RNAs comprising approximately 22 nucleotide sequences. Extracellular miRNAs are transported to recipient cells through exosomes, small vesicles, apoptotic bodies, and base pairs with the 3′untranslated region of the target mRNA to inhibit its transcription and translation (Bartel, 2004; Ha and Kim, 2014; Saliminejad et al., 2019). LncRNA is a molecule longer than 200 nucleotides that does not encode protein (Kopp and Mendell, 2018; Nair et al., 2020). Recent studies have found that lncRNAs participate in biological processes, such as the proliferation and differentiation of tumor cells, by regulating transcription and translation (Gao et al., 2020; Neve et al., 2021; Statello et al., 2021). lncRNAs regulate tumor development by exerting competing endogenous RNA actions through sponge miRNAs (Xu et al., 2018; Logotheti et al., 2020; Wan et al., 2020). Because circRNAs are stable and unaffected by exonucleases, they are more suitable diagnostic and prognostic markers than lncRNAs and miRNAs. Moreover, circRNAs have other biological functions; for example, in the nucleus, they can participate in transcriptional regulation and regulate gene expression. In the cytoplasm, they can be adsorbed by sponges and interact with RNA-binding proteins to promote or suppress tumors (Patop et al., 2019).

Bladder, prostate, and kidney cancers are the three most common malignancies of the urological system. A statistical analysis of Globocan 2008 and Global Cancer Statistics 2018 with a 10-year difference found that prostate and bladder cancers were among the top 10 cancers worldwide, with a high growth rate of >35% (Ferlay et al., 2010; Bray et al., 2018). Although several treatment strategies are available, patients with advanced disease usually have poor prognosis and low survival rate because of treatment insensitivity and susceptibility to recurrence. Therefore, it is necessary to develop novel diagnostic and therapeutic methods.

In recent years, the role of ncRNAs in human malignancies has been extensively studied. However, the role of ferroptosis-associated ncRNAs in common urological tumors has not been systematically described. This article reviews the latest progress in ncRNAs in common urological tumors, especially the regulation of ferroptosis by ncRNAs, which affects the occurrence, progression, treatment, drug resistance, and prognosis of tumors.

## 2 Overview of ferroptosis

**FIGURE 1:**
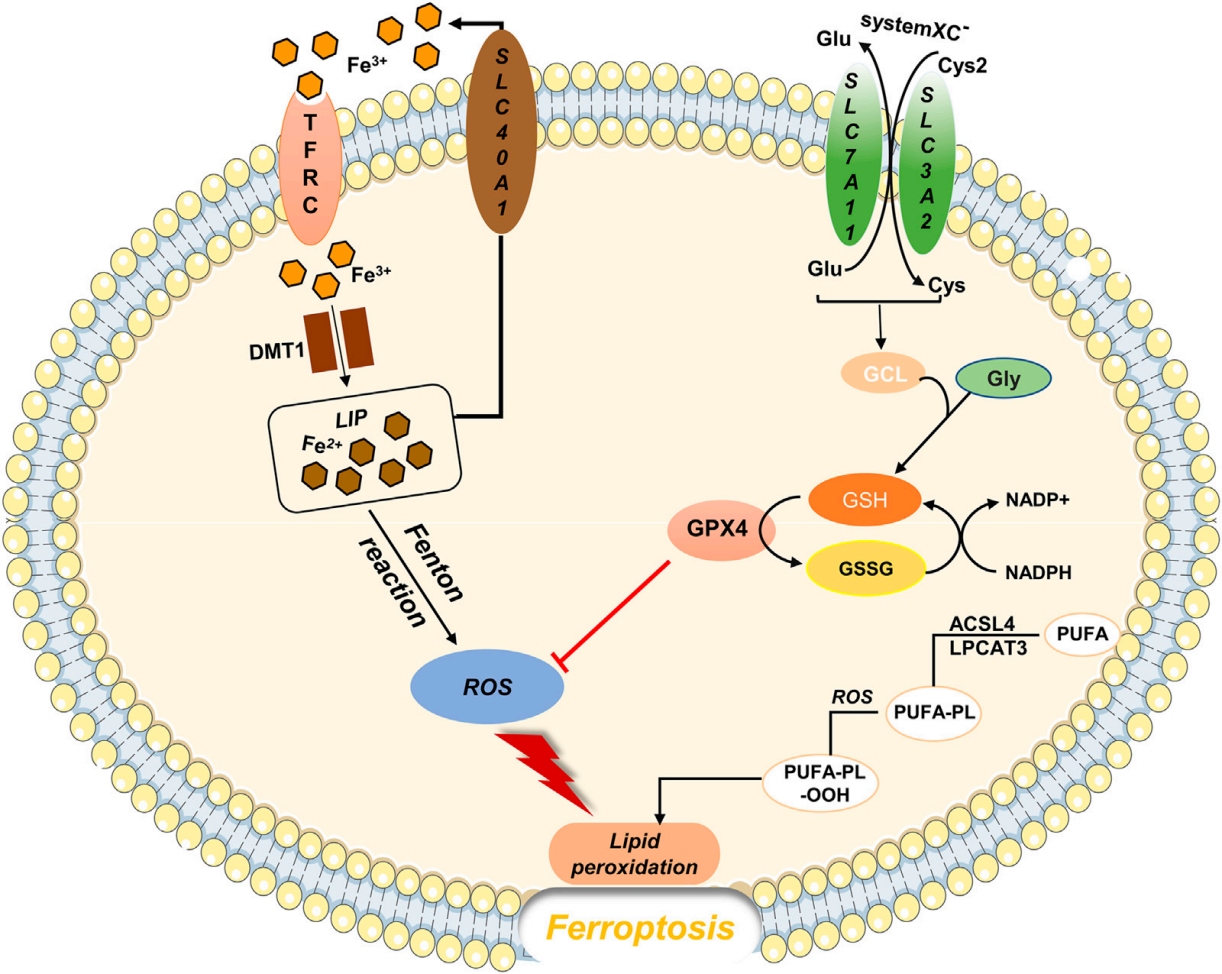
Regulatory substances and molecular mechanisms of ferroptosis The solute transporter protein SLC7A11 and transmembrane glycoprotein SLC3A2 form the cystine/glutamate transporter system (system XC-), which is embedded on the surface of the cell membrane and mediates the reverse exchange of cystine and glutamate. Cysteine is a key substrate for GSH synthesis. When GSH is depleted, GPX4 activity reduces, and the ROS generated by Fe through the Fenton reaction cannot be reduced, causing lipid peroxidation and oxidative stress, which leads to ferroptosis.

In 2012, Dixon proposed a mode of cell death that is morphologically, biochemically, and genetically distinct from apoptosis, necrosis, autophagy (Dixon et al., 2012). This form of cell death is referred to as ferroptosis, according to its characteristics: iron- and ROS-dependent cell death (Figure 1). Ferroptosis is typically characterized by an increase in the intracellular free Fe2^+^ content, which generates large amounts of ROS through the Fenton reaction. When ROS accumulation exceeds the reduction capacity of glutathione (GSH) or glutathione peroxidase (especially GPX4), it causes peroxidation of polyunsaturated fatty acids in the lipid membrane and disruption of cell membrane or plasma membrane structure and function, triggering ferroptosis (Yang et al., 2014; Kagan et al., 2017). Microscopically, mitochondrial wrinkling can be observed, mitochondrial cristae are reduced or disappear, and the mitochondrial membrane density increases (Gao et al., 2019; Gan, 2021). However, no cytoplasmic shrinkage or nuclear fragmentation is observed (Galluzzi et al., 2018).

## 3 Ferroptosis-associated ncRNAs and urological tumors

In recent years, an increasing number of studies have shown that ferroptosis-related ncRNAs play an important role in lung cancer (Song et al., 2021), gastrointestinal tumors (Zhang et al., 2020b; Zhu et al., 2020), liver cancer (Bai et al., 2020), breast cancer (Zhang et al., 2021a), glioma (Yang et al., 2021), acute leukemia (Wang et al., 2021) and other malignancies and may be potential diagnostic markers or therapeutic targets for tumors. Therefore, in this review, we describe the regulatory roles of ferroptosis-associated ncRNAs in common urological malignancies (Table 1). Moreover, we propose some challenges in the clinical applications of ferroptosis-associated ncRNAs. In our opinion, a systematic discussion of the regulatory patterns of ferroptosis-related ncRNAs in urological malignancies is extremely beneficial for exploring the prospects of tumor therapy, overcoming resistance to malignancy treatment, and improving patients’ quality of life.

**TABLE 1:** Overview of the role of ferroptosis-associated ncRNAs in urological malignancies.

Cancer type	Ferroptosis-associated ncRNA	Target	Influence to Target	Potential clinical application	Ref
Prostate cancer	miR-15A	GPX4	Down	Treatment and Prognosis	Xu et al. (2022)
	LncRNA OIP5-AS1	SLC7A11	Up	Treatment and Prognosis	Zhang et al. (2021b)
	LncRNA PCAT1	SLC7A11	Up	Drug resistance	Jiang et al. (2022)
Bladder cancer	LncRNA RP11-89	Prom	Up	Development	Luo et al. (2021)
	CircRNA-ST6GALNAC6	HSBP1	Down	Development and Treatment	Wang et al. (2022)
Kidney cancer	miR-4735-3p	SLC40A1	Down	Development, treatment and prognostic	Zhu et al. (2022)
	miR-324-3p	GPX4	Down	Treatment	Yu et al. (2022)
	LncRNA SLC16A1-AS1	SLC7A11	Up	Treatment	Li et al. (2022)

### 3.1 Ferroptosis-associated ncRNAs and bladder cancer

Extensive evidence suggests that ncRNAs are involved in regulating multiple processes in bladder cancer, particularly the tumor microenvironment (TME), drug resistance, and ferroptosis.

#### 3.1.1 NcRNAs and bladder cancer

Bladder cancer has one of the highest mutation rates among urological malignancies (Alexandrov et al., 2013; Tran et al., 2021). According to the 2020 global cancer statistics survey, new patients with bladder cancer account for approximately 3.0% of all patients with tumor, and the mortality rate is approximately 2.1% (Kaufman et al., 2009; Sung et al., 2021).

A close correlation between ncRNAs and cancer was observed in 2002, when Calin et al. found that deletion or downregulation of miR15 and miR16 genes, located on chromosome 13q14, were present in approximately 68% of chronic lymphocytic leukemia (CLL)cases (Calin et al., 2002). In 2007, it was discovered that 10 miRNAs, including miR-223 and miR-26b, were significantly upregulated in bladder cancer compared to normal tissues (Gottardo et al., 2007). Similarly, in 2009, Lin et al. found not only elevated miRNAs but also the downregulation of 38 miRNAs, including miRNA-143, miRNA-145, miRNA-125a, in bladder cancer tissues (Lin et al., 2009). With further research, there is evidence that abnormal expression of ncRNAs in tumors is associated with tumor progression, metastasis, and recurrence (Du et al., 2013; Iyer et al., 2015; Vo et al., 2019). For example, high expression of circRIP2 in bladder cancer is negatively correlated with the stage, grade, metastasis, and prognosis of bladder cancer. Further studies found that circRIP2 can increase Tgf-β2 levels in bladder cancer tissues by sponging miR-1305 and inducing tumor progression and epithelial–mesenchymal transition through the Tgf-β2/smad3 pathway (Su et al., 2020).

##### 3.1.1.1 Regulation of ncRNAs on TME in bladder cancer

The TME plays an important role in tumor migration and invasion, immunosuppression, and tumor drug resistance (Vitale et al., 2019). Tumor-associated macrophages (TAMs) are invaluable components of the TME. Bladder cancer-derived exosomal microRNA 21 (miR21) enhances STAT3 expression by inhibiting phosphatase activation in the PI3K/AKT signaling pathway and promotes M2 phenotype polarization in macrophages (Lin et al., 2020). This suggests that exosomal miR21 can promote bladder cancer migration and invasion by polarizing TAMs.

##### 3.1.1.2 Regulation of bladder cancer by ncRNAs

In addition, some ncRNAs act as tumor suppressors to inhibit tumor proliferation and invasion by regulating complex signaling networks. MicroRNA-139-5p, a mesenchymal stem cell-derived exosome, can promote bladder cancer cell apoptosis by silencing polycomb repressive complex 1 (PRC1) in bladder cancer, thereby inhibiting tumor cell proliferation and migration (Jia et al., 2021). The tumor suppressive effect of miRNA-139-5p may be a novel direction for tumor therapy. Similarly, miRNA-143 transfected into T24 and EJ cells was shown to inhibit tumor cell proliferation, suggesting that miRNA-143 may be an important tumor suppressor in bladder cancer (Lin et al., 2009).

##### 3.1.1.3 Immunotherapy for bladder cancer

Immunotherapy for bladder cancer has been performed since the 1970’s, from intracavitary infusion of *Bacillus* Calmette-Guerin to immune checkpoint inhibitors in recent years. A history of over 50 years suffices to demonstrate the importance of the immune microenvironment in the treatment of bladder cancer. A recent clinical study demonstrated the safety and feasibility of a combination of Cabozantinib and Programmed Death Receptor-1 (CaboNivo) in the treatment of bladder cancer (Apolo et al., 2020). Although there are various treatments for bladder cancer, the high recurrence rate and susceptibility to metastasis of bladder cancer are still challenging issues. Therefore, it is important to search for more effective treatment methods to improve the prognosis of bladder cancer and ultimately improve the quality of life of patients.

#### 3.1.2 Regulation of ferroptosis in bladder cancer by ncRNAs

Ferroptosis is a novel iron-dependent form of RCD caused by ROS accumulation and lipid peroxidation of lipid membranes, leading to cell death (Dixon et al., 2012; Sun et al., 2022a). ncRNAs play an important role in gene expression at the epigenetic, transcriptional, and post-transcriptional levels through interaction with different DNAs, RNAs, or proteins. With advancements in research, the induction of ferroptosis in tumor cells has become an effective and feasible treatment, especially for malignant tumors that are not sensitive to conventional treatments (Hassannia et al., 2019; Liang et al., 2019; Sui et al., 2022). This section provides a systematic description of the role of ferroptosis-related ncRNAs in bladder cancer, hoping that it will be useful in the diagnosis and treatment of bladder cancer.

Recent studies on ferroptosis-related ncRNAs in bladder cancer may help explore the pathogenesis and therapeutic strategies of human malignancies. MicroRNA-129-5P (miR-129-5P) is a key regulatory gene in malignancies of the urinary system. For example, overexpression of miR-129-5P in clear cell renal cell carcinoma (CCRCC) inhibits the function of sialophorin, which eliminates the promotion effect of SPN on cell proliferation and invasion, while simultaneously inducing the cell cycle arrest of tumor cells in G0/G1 phase (Gao et al., 2021). Similarly, high expression of miR-129-5P in prostate cancer downregulates the level of a serine/threonine-specific protein kinase (CAMK2N1), and targeting miR-129-5P sensitizes patients with prostate cancer to the chemotherapy drug docetaxel (Wu et al., 2020). Furthermore, miR-129-5p is sponged by the highly expressed lncRNA RP11-89 in bladder cancer cells, which induces proliferation and metastasis of bladder cancer cells and inhibits cell cycle arrest (Luo et al., 2021). Meanwhile, lncRNA RP11-89 can upregulate prom2 expression level through the prominin2-multivesicular body (MVB)-exosome-ferritin pathway, resulting in increased resistance to ferroptosis in bladder cancer (Brown et al., 2019; Luo et al., 2021). The mechanism is that prominin2 protein can transport iron out of cells through the MVB/exosome pathway, reducing the accumulation of intracellular iron and preventing ferroptosis. This discovery suggests that lncRNA RP11-89 may be a key gene for the proliferation and metastasis of bladder cancer, and the regulation of ferroptosis by targeting ncRNAs can aid in the development of different therapeutic directions for bladder cancer.

In addition, recent studies have shown that circular RNA can influence tumor proliferation and invasion (Memczak et al., 2013; Lei et al., 2020; Zang et al., 2022). For example, overexpression of circRNA-ST6GALNAC6 can promote ferroptosis in bladder cancer cells (Wang et al., 2022). CircRNA-ST6GALNAC6 binds to the N-terminal phosphorylation site (SER-15) of the heat shock protein family member HSBP1. It leads to the downregulation of HSPB1 phosphorylation, inhibits the activation of the HSPB1/p38 MAPK signaling pathway, and increases the susceptibility of tumor cells to ferroptosis.

Furthermore, ferroptosis-related ncRNAs participate in the regulation of tumor drug resistance. Cisplatin is a common clinical treatment for tumors, including bladder cancer; however, deriving benefit from it is often difficult for many patients with cancer because of its drug resistance (Galluzzi et al., 2012; Browning et al., 2017). Recent studies have shown that the solute transporter family member SLC7A11 is not only a key component of the cystine/glutamate antiporter (XC system) but also an important regulator of bladder cancer resistance to cisplatin (Lo et al., 2008; Lewerenz et al., 2013). Overexpression of miR-27a can negatively regulate SLC7A11 protein and intracellular GSH levels, which leads to the re-sensitization of drug-resistant tumor cells (Drayton et al., 2014). Similar results have also been observed in ovarian cancer. Notably, a certain dose of sulfasalazine can eliminate the cisplatin resistance of SLC7A11-induced bladder cancer. In addition, Hou et al. reported that bioinformatics was used to screen 11 lncRNAs associated with poor prognosis of bladder cancer and to construct a prognostic model that may improve the treatment of patients with bladder cancer (Hou et al., 2022). In summary, the application of ferroptosis-related ncRNAs in bladder cancer has promising prospects.

With an in-depth study of the disease, more molecular mechanisms of its pathogenesis and key regulatory proteins have been understood. These findings are beneficial for the individualized treatment of patients with cancer and to improve their quality of life.

### 3.2 Ferroptosis-associated ncRNAs and prostate cancer

The incidence of prostate cancer is second only to lung cancer among male malignant tumors, with an estimated annual death rate of approximately 374,000 people (Sung et al., 2021). Although the treatment strategy for prostate cancer has changed from single surgery, androgen deprivation therapy, to the combined application of chemotherapy, endocrine therapy, and immunotherapy, the survival and prognosis of patients are still not optimistic (Karantanos et al., 2015; Roubaud et al., 2017; Teo et al., 2019). Therefore, identification of novel diagnostic markers and therapeutic strategies is necessary.

Some studies have shown that ferroptosis-related ncRNAs may be potential therapeutic targets or diagnostic markers. For example, a recent study showed that the combined application of ferroptosis inhibitors and second-generation anti-androgens could significantly inhibit the growth and migration of prostate cancer cells, providing a novel strategy for the treatment of prostate cancer (Ghoochani et al., 2021). Likewise, ncRNAs in prostate cancer tissue or serum have received considerable attention in recent years as potential biomarkers of tumors. The lncRNA PCAT1 in prostate cancer upregulates androgen-responsive gene levels through the recruitment of androgen receptor and lysine-specific demethylase 1, leading to the growth and proliferation of prostate cancer cells (Guo et al., 2016). In addition, many differentially expressed ncRNAs, which are the best candidates for non-invasive biomarkers, were found in the plasma or urine of patients, which are the best candidates for non-invasive biomarkers (Hua et al., 2019).

Numerous studies have suggested that multiple signaling pathways, including vascular endothelial growth factor, TGF-β, and JAK/STAT, are involved in the development of prostate cancer and may be regulated tightly by ncRNAs. The transforming growth factor (TGF)-β signaling pathway is one of the most widely studied signaling networks and plays an important regulatory role in biological behaviors, such as growth and development, invasion, and metastasis of tumor cells. When TGF-β initiates the Smad signaling pathway, TGF-β first binds to TGF-β receptor 2 (TβRII) on the cell membrane and recruits TGF-β receptor 1 to form a complex of TGF-β-TβRII-TβRI type receptors. Subsequently, TβRI in the complex undergoes a conformational change and phosphorylates Smad2 and Smad3 proteins. Activated Smad2 and Smad3 combine with Smad4 to translocate to the nucleus to bind DNA and regulate the expression of target genes (Guo and Kyprianou, 1998). The expression of the TGF-β signaling pathway in prostate cancer has a dual role. In the early stage of the disease, it is a tumor suppressor that resists tumor proliferation; in the late stage, not only is its tumorigenic effect enhanced, but it also promotes tumor metastasis (Ware, 1993; Lee et al., 1999). Therefore, intervention in the TGF-β/Smad signaling pathway at the early stage of the disease can help improve the prognosis and therapeutic effect of the disease. MicroRNA-15A (mi15A) has multiple roles in prostate cancer cells. On the one hand, as a tumor suppressor, miR-15A binds to the 3′non-coding end (3′-UTR) of Smad3 and inhibits its activity. Ultimately, miR-15A affects the TGF-β signaling pathway and inhibits the invasion and metastasis of prostate cancer cells (Bonci et al., 2008; Jin et al., 2018). On the other hand, recent studies have shown that miR-15A can interact with the 3′-UTR of GPX4, a key protein in iron death, to influence GPX4 protein expression. Moreover, transfection of prostate cancer cells with miR-15A mimics or Si-GPX4 resulted in a significant increase in intracellular Fe^+^ and ROS levels. These findings suggest that targeting miR-15A can induce ferroptosis in prostate cancer cells by downregulating GPX4 protein levels (Xu et al., 2022).

Although the precise cause of prostate cancer development is not yet thoroughly understood, current research indicates that genetic and environmental factors induce prostate cancer development (Deutsch et al., 2004). Bordini et al. found that iron accelerates oxidative stress and cell death in prostate cancer cells and contributes to the efficacy of anti-androgen therapy (Bordini et al., 2020). In addition, a recent study showed that long-term exposure to Cd promotes cancer cell growth and inhibits ferroptosis (Zhang et al., 2021b). Mechanistically, lncRNA OIP5-AS1 suppressed ferroptosis in cadmium-exposed tumor cells by competitively binding to miR-128-3P and increasing the expression level of SLC7A11, a critical protein for ferroptosis.

Resistance to the chemotherapy drug docetaxel (DTX) in patients with prostate cancer is one of the major causes of poor survival in most patients (Virgo et al., 2021). Recent studies have shown that ferroptosis-associated ncRNAs are also involved in the mechanism of drug resistance in prostate cancer. For example, lncRNA PCAT1, activated by the transcription factor ap-2γ, activates the expression of SLC7A11 by interacting with the oncogene myc/miR-25-3p, which blocks DTX-induced ferroptosis and enhances chemotherapy resistance (Jiang et al., 2022). In addition, some predictive models suggest that ferroptosis-related lncRNAs are associated with biochemical recurrence, immune invasion, and the TME in prostate cancer (Liu et al., 2022a; Feng et al., 2022).

Ferroptosis-related ncRNAs act as pivotal regulators at different stages of various diseases, including cancer. Research on ferroptosis-related ncRNAs as prostate cancer biomarkers, drug resistance, and treatment strategies is still at an early stage but has shown great research value and broad application prospects.

### 3.3 Ferroptosis-associated ncRNAs and kidney cancer

Kidney cancer, a malignant tumor originating in the renal parenchyma, is a major disease that poses a serious threat to human health. Despite the availability of multiple therapeutic strategies, it is still necessary to explore novel therapeutic approaches and tumor markers. Recent studies have shown that tumor cells are more sensitive to ferroptosis than normal cells, particularly renal cancer cells (Yang et al., 2014; Zou et al., 2019; Green et al., 2022). Therefore, in-depth studies on the role of ferroptosis-associated ncRNAs in kidney cancer may be meaningful for the discovery of novel tumor diagnostic markers and potential therapeutic strategies (Zhang et al., 2019; Lei et al., 2022; Li et al., 2022; Zhu et al., 2022).

Abnormal expression of some ncRNAs in cancer is closely related to cell proliferation, invasion or chemotherapy resistance. Li et al. (2021) discovered that the upregulation of microRNA-153-5p is closely related to the poor prognosis and metastasis of CCRCC, and the knockdown of the miR-153-5p gene can significantly inhibit the proliferation and invasion of tumor cells. Furthermore, miRNA-424-5p inhibits the proliferation and metastasis of CCRCC cells and reduces cell viability (Kalantzakos et al., 2021). Similarly, ectopic expression of MicroRNA-200b inhibits invasion and metastasis of CCRCC (Li et al., 2019). Aberrant expression of some ncRNAs, such as miR-223-3p, miR-543, miR-186, and lncHILAR, has also been observed in CCRCC and is closely associated with disease progression and prognosis (Chen et al., 2018; Xiao et al., 2019; Guo et al., 2020; Hu et al., 2021).

Recently, the role of ferroptosis-associated ncRNAs in kidney cancer has attracted considerable interest from researchers. For example, ncRNAs can affect ferroptosis by regulating the expression of SLC40A1. As a member of the solute transporter (SLC) family, SLC40A1 is currently the only key protein that regulates iron transport in mammals and plays an important role in maintaining the balance of iron in and out of cells (Hentze et al., 2010). Mechanistically, miR-4735-3p binds to the 3′-terminal non-coding region (3′-UTR) of SLC40A1 to repress its expression, leading to increased levels of intracellular iron loading, ROS, and lipid peroxidation (Zhu et al., 2022). This, in turn, leads to ferroptosis and tumor suppression in renal cancer cells.

Additionally, ferroptosis-related ncRNAs explain the mechanism of traditional Chinese medicine in the treatment of kidney cancer. Icariside II (ICS-II) is a flavonoid extracted from the Chinese herb Epimedium that has attracted considerable attention because of its anti-tumor activity (Liu et al., 2020; Atanasov et al., 2021).

This study found that after ICS-II treatment, the expression of miR-324-3p was upregulated, which inhibited the expression of GPX4 protein and induced ferroptosis in renal cancer cells. Interestingly, normal cells are not affected (Yu et al., 2022). ICS II downregulates GPX4 levels in a p53-independent manner to induce ferroptosis in tumor cells, which highlights the potential value of traditional Chinese medicine in the treatment of tumors. Not only are miRNAs important regulatory genes in the occurrence and metastasis of kidney cancer, lncRNAs are also critical for the treatment of kidney cancer. Researchers have found that the lncRNA SLC16A1-AS1 forms an RNA–protein complex with transcription factor E2F1, which enhances metabolic reprogramming and invasiveness of bladder cancer by promoting the expression of SLC16A1/MCT1 (Logotheti et al., 2020). In contrast, the highly expressed lncRNA SLC16A1-AS1 in CCRCC inhibits ferroptosis by sponge-adsorbing the tumor suppressor gene miR-143-3P and upregulating the expression of the key protein of ferroptosis-SLC7A11 (Li et al., 2022). In conclusion, the above study provides a novel perspective for the treatment of kidney cancer, i.e., silencing SLC16A1-AS1 induces ferroptosis in tumor cells by increasing miR-143-3P levels.

In addition, bioinformatics analyses offer guidelines to effectively investigate the role of ferroptosis-related ncRNAs in kidney cancer in the future. For example, risk assessment and diagnostic models based on five ferroptosis-associated lncRNAs by Shu et al. may help in the prognosis and diagnosis of kidney cancer (Shu et al., 2021). Furthermore, there are additional predictive models showing that ferroptosis-related lncRNAs are associated with changes in drug resistance, the TME, immune infiltration, immune escape, and TME in kidney cancer, which may help individualize treatment for patients (Bai et al., 2022; Liu et al., 2022b; Sun et al., 2022b; Chen et al., 2022; Yang et al., 2022; Zhou et al., 2022).

Targeting ncRNAs to regulate ferroptosis could be a novel strategy for the treatment of human malignancies, allowing for improved individualized treatment for patients with cancer.

## 4 Conclusion

**FIGURE 2:**
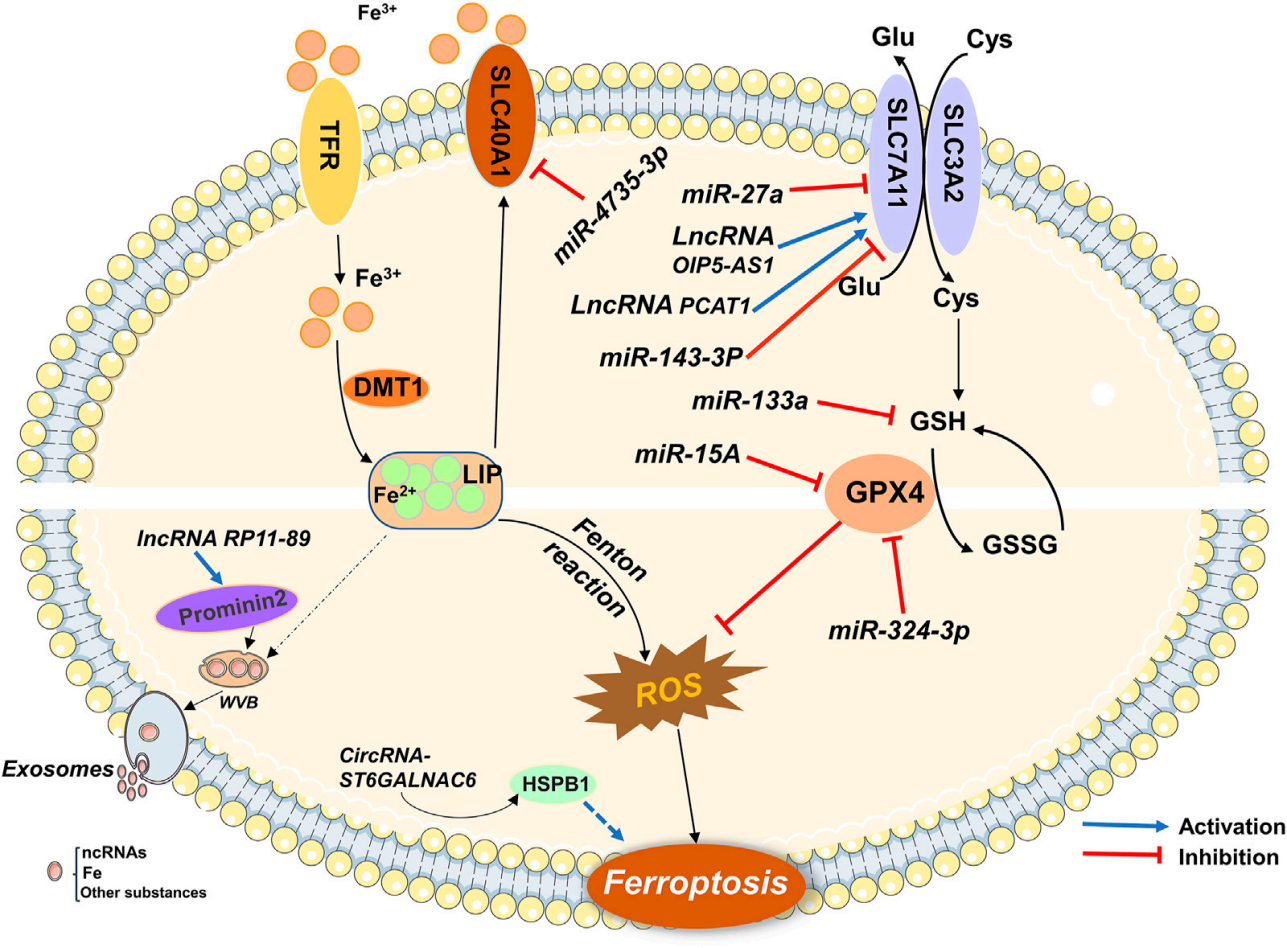
Ferroptosis-related ncRNAs participate in the regulation of urological malignancies.

As a new mode of regulatory cell death, ferroptosis plays a vital role in the occurrence, development, treatment, and prognosis of common malignant tumors in the urinary system. The role of ferroptosis-associated ncRNAs in urological malignancies has progressed. With advancements in research, a deep understanding of the pathogenesis of human malignancies has been obtained. Furthermore, ncRNAs have been shown to regulate urological malignancies through the ferroptosis pathway (Figure 2). Non-coding RNAs, marginal molecules that have been neglected in the past, are expected to affect biological behaviors, such as proliferation, differentiation, and metastasis, of tumor cells by regulating gene expression and become therapeutic targets and diagnostic markers for urinary system malignancies.

The divergent expression of ncRNAs in different urological tumors provides a theoretical basis for precise individualized treatment of patients with tumor. However, many challenges remain in the clinical application of ferroptosis-associated ncRNAs in urological malignancies. First, the specific regulatory mechanisms of ferroptosis-related ncRNAs in urological malignancies are not yet fully understood. Therefore, in-depth studies are required to determine its clinical application. Second, there is also a crucial issue that ncRNAs regulate cell death in a variety of ways, including ferroptosis, apoptosis, necrosis, and autophagy. Further investigation of the correlations between ncRNAs and ferroptosis, necrosis, and autophagy could deepen our understanding of the relationship between gene expression and cell death. However, the role of ferroptosis-associated ncRNAs in urological malignancies remains largely unexplored. This will undoubtedly be one of the key directions for future research.

We systematically described the roles of several ferroptosis-associated ncRNAs in common urological malignancies. These findings not only deepen our understanding of ncRNAs in urological malignancies but also highlight the prospects of ferroptosis-related ncRNAs in the development and treatment of urological malignancies. Overall, targeting ncRNAs to regulate ferroptosis may help develop novel therapeutic strategies to overcome the current dilemma in the treatment of urological malignancies.

## Abbreviations

CCRCC, clear cell renal cell carcinoma; circ RNA, circular RNA; lnc RNA, long non-coding RNA; miRNA, microRNA; ncRNA, non-coding RNA; RCD, regulated cell death; GSH, Glutathione; GPX4, glutathione peroxidase 4; SLC3A2, solute carrier family 3 member2; SLC7A11, solute carrier family 7 member11; system XC^-^, cystine/glutamate transporter system.
